# How commercial and non-commercial swine producers move pigs in Scotland: a detailed descriptive analysis

**DOI:** 10.1186/1746-6148-10-140

**Published:** 2014-06-25

**Authors:** Thibaud Porphyre, Lisa A Boden, Carla Correia-Gomes, Harriet K Auty, George J Gunn, Mark EJ Woolhouse

**Affiliations:** 1 Centre for Immunity, Infection and Evolution, University of Edinburgh, King’s Buildings, Edinburgh, UK; 2 Institute of Comparative Medicine, Faculty of Veterinary Medicine, Bearsden, University of Glasgow, Glasgow, UK; 3 Epidemiology Research Unit, SRUC, Drummondhill, Stratherrick Road, Inverness, UK

**Keywords:** Livestock movement, Risk of infection, Pig, Contingency planning

## Abstract

**Background:**

The impact of non-commercial producers on disease spread via livestock movement is related to their level of interaction with other commercial actors within the industry. Although understanding these relationships is crucial in order to identify likely routes of disease incursion and transmission prior to disease detection, there has been little research in this area due to the difficulties of capturing movements of small producers with sufficient resolution. Here, we used the Scottish Livestock Electronic Identification and Traceability (ScotEID) database to describe the movement patterns of different pig production systems which may affect the risk of disease spread within the swine industry. In particular, we focused on the role of small pig producers.

**Results:**

Between January 2012 and May 2013, 23,169 batches of pigs were recorded moving animals between 2382 known unique premises. Although the majority of movements (61%) were to a slaughterhouse, the non-commercial and the commercial sectors of the Scottish swine industry coexist, with on- and off-movement of animals occurring relatively frequently. For instance, 13% and 4% of non-slaughter movements from professional producers were sent to a non-assured commercial producer or to a small producer, respectively; whereas 43% and 22% of movements from non-assured commercial farms were sent to a professional or a small producer, respectively. We further identified differences between producer types in several animal movement characteristics which are known to increase the risk of disease spread. Particularly, the distance travelled and the use of haulage were found to be significantly different between producers.

**Conclusions:**

These results showed that commercial producers are not isolated from the non-commercial sector of the Scottish swine industry and may frequently interact, either directly or indirectly. The observed patterns in the frequency of movements, the type of producers involved, the distance travelled and the use of haulage companies provide insights into the structure of the Scottish swine industry, but also highlight different features that may increase the risk of infectious diseases spread in both Scotland and the UK. Such knowledge is critical for developing more robust biosecurity and surveillance plans and better preparing Scotland against incursions of emerging swine diseases.

## Background

The pig industry in Scotland is a small but very well organised industry which focuses on assured production of high quality farrow-to-finish pigs, with a large proportion of outdoor herds. The Scottish pig sector comprises over 321,000 pigs [1] and accounts for nearly 7% of the UK pig herd [2]. The industry contributes about 2.8% of the Scottish Agricultural Output (approximately £77.8 million) and is associated with exports worth £3 million [3]. However, pig prices have not kept up with the cost of production and the Scottish pig industry has declined in size by more than 34% in the last decade. In comparison, the number of cattle and sheep farms decreased by 9 and 14% in the same period [1]. As such, the industry is probably more vulnerable than other livestock sectors to threats such as exotic or emerging diseases that, if introduced into the pig population, would undoubtedly have major economic consequences.

In addition to commercial producers, Scotland has a number of small pig producers, including “backyard” producers, crofters and small holdings. Although this sector of the industry may represent a small proportion of the pig industry in term of the numbers of animals reared, more than two thirds of the pig producers in Scotland report less than 10 pigs (69%, Scottish Agricultural census 2011). Evidence from other countries have shown considerable interaction between small pig producers and commercial producers [4], whether directly, through the trade of live animals, or indirectly, through the use of slaughterhouses, market facilities and haulage services. In addition, they represent the least-well regulated sectors of the industry where biosecurity may be sub-optimal [5,6]. As such, their impact on disease transmission during outbreaks may have been underestimated [7-9]. To ensure early detection, management and control of any future emerging infectious disease outbreak, knowledge of the degree of interconnection as well as on spatial and temporal aspects of animal movements between non-commercial and commercial producers is therefore critical. However, such information is rare, as most information collected, is based on large commercial farms [5,7,10] or is obtained from countries where non-commercial pig farming is limited [11-14]. For instance, 5%, 11%, 15% and 19% of registered farms report less than 10 pigs in Denmark, Netherlands, Belgium and Sweden, respectively [15].

The Scottish Livestock Electronic Identification and Traceability database (ScotEID), which was introduced in November 2011, is a unique resource for better understanding the inter-connectivity of pig movements between all types of producers. Under Scottish [16] and European legislation [17], all pig keepers moving animals are not only required to register online with ScotEID and electronically record any movements ahead of time but also report details of any haulage company employed for the transport. The objectives of this research were threefold. First, to use the ScotEID database to characterize movements between pig premises in Scotland, specifically, quantifying the amount, frequency and distance of movements between pig premises. Second, to investigate different movement patterns in the Scottish pig industry which may affect the risk of disease spread. In particular, we focus on the role of small pig producers. Finally, acknowledging that disease may spread between farm premises through contaminated livestock vehicles [18,19], we aim to explore the frequency of usage of livestock hauliers within the Scottish pig industry.

## Results

### Movement numbers and premises types

Overall, 23,169 batches of pigs were recorded moving animals between 2382 known unique premises during the 17-month study period. A description of the numbers of movements, stratified by types of pig producer (according to origin premises), are described in Table 1. The number of movements originating and departing from premises of different producer types is described in Table 2. In total, 14,105 (61%) of all pig movements terminated at slaughterhouses. Of these, 13,570 (96%) were direct movements between a pig producer and a slaughterhouse; the remaining 535 (4%) moved from a market to a slaughterhouse.

**Table 1:** Descriptive statistics of outgoing pig movements

**Property**	**Total**^**a**^	**Small producers**	**Non assured**	**Professionals**
Number of active^b^ premises	2382	1755	285	290
Number of batches sent (% to slaughter)	23,169 (61%)	3888 (60%)	6277 (69%)	12,237 (56%)
Number of animal sent (% to slaughter)	1,993,396 (47%)	12,569 (56%)	455,467 (52%)	1,434,441 (47%)
**Movement to slaughter**				
Median batch size (Q1-Q3)	35 (4–108)	2 (2–4)	20 (4–80)	80 (33–164)
Max batch size	368	50	235	368
% of movements with >100 animals	26%	0%	20%	41%
Median Euclidean distance in km (Q1-Q3)	95 (35–194)	33 (18–59)	101 (36–153)	129 (56–199)
Max Euclidean distance in km	724	593	718	724
% of movements of >100 km	49%	10%	51%	58%
Number of cross-border movements	3144	141	1048	1529
% of cross-border movements going South	89%	70%	80%	94%
Number of cross-border animals	251,206	747	62,137	169,985
% of cross-border animals going South	92%	83%	94%	90%
**Movement to other premises types**				
Median batch size (Q1-Q3)	25 (5–197)	2 (2–4)	30 (5–199)	73 (12–225)
Max batch size	1934	50	1752.0	1234
% of movements with >100 animals	34%	0%	32%	45%
Median Euclidean distance in km (Q1-Q3)	35 (15–96)	27 (11–58)	35 (14–182)	36 (17–95)
Max Euclidean distance in km	762	762	709	696
% of movements of >100 km	24%	13%	31%	24%
Number of cross-border movements	1246	179	401	593
% of cross-border movements going South	84%	52%	91%	88%
Number of cross-border animals	312,078	862	65,890	175,494
% of cross-border animals going South	96%	70%	90%	98%

^a^All premises, including markets, shows ground, ferry terminals, slaughterhouses and farms.

^b^Active premises defined as those that sent or received pigs during the study period.

**Table 2:** Description of movements of at least 1 pig from January 2012 to May 2013

		**Movements from**			**Movements to**		
	**N**^**a**^	**Total**	**With haulier**	**%**^**b**^	**Total**	**With haulier**	**%**
**Premises type**							
Small-pig producer	1755	3888	108	3%	1927	211	11%
Non-assured commercial producers	285	6277	3550	57%	1232	798	65%
QHA producers	290	12237	9973	81%	4019	3601	90%
Slaughterhouse	29	2	0	0%	14105	8448	60%
Market	13	682	259	38%	1858	892	48%
Showground	8	20	0	0%	22	0	0%
Ferry collection centres	2	63	62	98%	6	2	33%
**Scottish Regions**							
Highlands and Islands	677	2690	821	31%	2058	274	13%
Mid Scotland and Fife	185	1464	562	38%	1311	166	13%
Scotland Central Belt	229	1994	901	45%	5134	3904	76%
South Scotland	428	3538	1810	51%	2337	841	36%
East Scotland	514	12872	9618	75%	8471	5639	67%
**English Regions**							
Yorkshire and The Humber	70	30	21	70%	371	351	95%
North East England	105	175	32	18%	1501	1362	91%
North West England	83	256	84	33%	660	470	71%
West Midlands	13	5	0	0%	31	22	71%
East Midlands	21	46	27	59%	685	458	67%
East of England	29	12	3	25%	585	460	79%
South East England	5	0	0	0%	7	1	0%
South West England	10	6	4	67%	7	2	29%
**Northern Ireland**	-	63	62	98%	6	2	33%
**Wales**	8	8	3	38%	4	0	0%
**Other countries**	-	10	4	40%	1	0	0%

Movements were classified by location type and region. Also shown are movements of at least 1 pig when a professional haulier is used for transportation.

^a^Number of premises.

^b^Proportion of movements using a haulage company.

**Figure 1:**
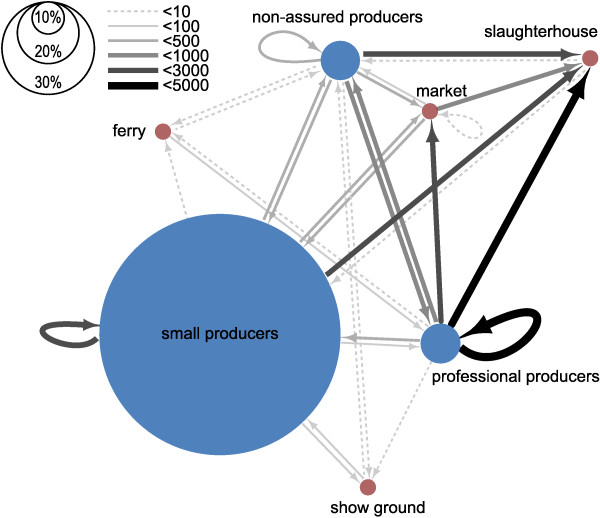
**Connectivity within and between premises types through pig movements as recorded in ScotEID between January 2012 and May 2013.** Each premises type is represented by a circle; arrows represent the movement of pigs. The width of the line is proportional to the number of batches moved between premises types. The size of the circles for producers is proportional to the number of producers per producer type.

The interconnectivity of pig movements between all types of premises (including markets, show grounds, ferry collection centres in addition to the three classifications of pig producers) is shown in Figure 1. As expected, the network was dominated by movement to slaughterhouse. Even though small producers outnumber commercial producers (professional or non-assured), 83% of these movements to slaughter were made from professionals (51%) or from non-assured commercial producers (32%) (Table 1, Figure 1). When movements to slaughterhouses were excluded, most movements occurred directly between producers (n = 6951, 77%), or between a producer and a market collection centre (n = 1995, 22%), but a small number of movements also occurred between producers and shows (n = 42, <1%). Although most movements occurred between producers of similar type, movements between producers of different types also occurred (Figure 1). For instance, 13% and 4% of movements from professional producers were sent to a non-assured commercial producer or to a small producer, respectively; whereas 43% and 22% of movements from non-assured commercial farms were sent to a professional or a small producer, respectively. In contrast, only 8.6% of movements from small producers were sent to commercial producers (7.3% to non-assured producers and 1.3% to professionals).

The calculated values of the E-I (external - internal) index, a measure of group assortativity, between producer types are described in Table 3. Professional producers and small producers showed tendencies to strictly receive (−0.57 and −0.26 respectively) or send (−0.54 and −0.79 respectively) pigs to producers with the same producer type, whereas non-assured commercial producers tended to trade (0.52 and 0.34 for sending and receiving, respectively) with professional or small producers rather than other non-assured commercial producers. Looking at weekly E-I indices (Table 3), mean estimates were similar to what was calculated over the whole study period, but variations occurred, particularly for movements received by small and non-assured professionals.

**Table 3:** Pig movements assortativity between regions, and between farm types for all premises involved in ScotEID between January 2012 and May 2013

**Groups**	**Emit E-I index overall**	**Emit E-I index weekly**	**Receive E-I index overall**	**Receive E-I index weekly**
**Farm Type**^**a**^				
Professional producers	−0.54	**−0.53** (−0.72 – −0.38)^b^	−0.57	**−0.56** (−0.67 – −0.46)
Non assured commercial producers	0.52	**0.53** (0.42 – 0.67)	0.34	0.29 (0.09 − 0.46)
Small producers	−0.79	**−0.78** (−0.89 – −0.69)	−0.26	−0.24 (−0.43 – −0.1)
**Region**^**c **^**(non-slaughter)**				
Highlands and Islands	−0.37	−0.36 (−0.60 – –0.15)	−0.65	−0.64 (−0.88 – −0.44)
Mid Scotland and Fife	−0.22	−0.24 (−1.00 – 0.33)	0.18	0.09 (−0.14 – 0.43)
Scotland Central Belt	−0.04	0.05 (−0.25 – 0.43)	0.38	0.28 (−0.04 – 0.71)
South Scotland	0.22	0.17 (−0.06 – 0.41)	−0.08	−0.15 (−0.50 – 0.20)
East Scotland	−0.59	**−0.59** (−0.78 – −0.40)	−0.83	**−0.83** (−0.88 – −0.79)
**Region**^**d **^**(slaughter)**				
Highlands and Islands	−0.44	−0.41 (−0.57 – −0.29)	−0.98	−**0.98** (−1.00 – −1.00)
Mid Scotland and Fife	0.07	0.08 (−0.05 – 0.18)	−0.18	−0.19 (−0.38 – −0.07)
Scotland Central Belt	0.33	0.25 (0.00 – 0.55)	0.77	0.60 (0.30 – 0.88)
South Scotland	0.15	0.17 (0.07 – 0.26)	−0.33	**−0.32** (−0.42 – −0.24)
East Scotland	0.23	0.18 (−0.22 – 0.48)	−0.63	**−0.61** (−0.69 – −0.53)

Bold numbers show where the computed mean weekly External–internal (E-I) index of pig movement connections was considered significantly different from the non-assortativity hypothesis (i.e. when the confidence envelop around the mean, as defined by ±2SD, does not overlap zero).

^a^Estimates computed for movements involving agricultural holdings only.

^b^Mean and the Q1-Q3 range of the distribution of the computed E-I index for the 73 complete weeks recorded in the study period.

^c^Only Scottish regions are reported, estimates computed for movements involving agricultural holdings and gathering places.

^d^Only Scottish regions are reported, estimates computed for movements to slaughter only.

### Batch size

There were 1,993,396 animals represented by 23,169 movements. Of these, 933,634 (47%) pigs were moved to be slaughtered (Table 1). Of these 933,634 pigs, 669,718 (72%) came from professional producers, 209,037 (22%) came from non-assured commercial producers and 54,879 (6%) came from small pig producers (Table 1). Median batch size of slaughter and non-slaughter movements are described in Table 1. While small producers move significantly less pigs per batch (median 2, Q1-Q3 2–4), non-assured commercial producers and professional pig producers moved similar batch sizes.

### Timing of movements

**Figure 2:**
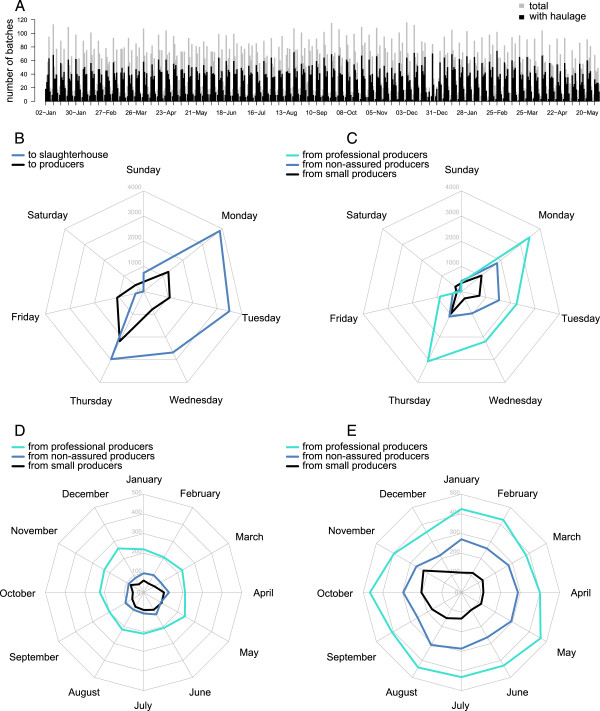
**Dynamics of pig movements as recorded in ScotEID between January 2012 and May 2013. (A)** Total number of batches of pigs moved daily during the study period, stratified by the use of a registered haulage company or not. Number of batches of pigs moved in each day of the week, stratified by the type of the destination **(B)** and type of the departure **(C)**. Number of batches of pigs moved to a producer **(D)** and to slaughter **(E)** in each month from January to December 2012, stratified by the type of the departure.

During the 17-month study period, there were 516 possible days of movement activity. On 514 days, at least one movement was recorded (i.e. there were only two days, Sunday 1st of January 2012 and Saturday 9th of February 2013, within the study period during which there were no movements recorded). Overall, a median of 46 (Q1-Q3: 15–71), 318 (Q1-Q3: 296–333) and 1364 (Q1-Q3: 1276–1459) movements were carried out each day, week and month, respectively; among which 13 (Q1-Q3: 7–18), 94 (Q1-Q3: 86–102) and 405 (Q1-Q3: 385–409) were made between producers. Looking at the broad contact rate between producers, small producers rarely moved pigs to another producer (mean 0.0048 movements/day, median 0.0019/day, range = 0.0019/day - 0.0504/day), whereas non-assured and professional producers moved pigs with a mean frequency of 0.0196/day (median 0.0058/day, range 0.0019 movements/day - 0.3760/day) and 0.0550/day (median 0.0194/day, range 0.0019/day - 0.6066/day), respectively. Similarly, there was a 10- to 20-fold difference in the rate of pigs sent to slaughterhouse from small producers in comparison with non-assured and professional producers. Indeed, small producers sent pigs to slaughter with a mean frequency of 0.0047/day (median 0.0019/day, range 0.0019 movements/day - 0.1105/day), whereas non-assured and professional producers sent pigs with a mean frequency of 0.0407/day (median 0.0155/day, range 0.0019 movements/day - 0.3779/day) and 0.0916/day (median 0.0494/day, range 0.0019/day - 0.9845/day), respectively.Figure 2A shows the total number of batches of pigs moved daily during the study period. There was a clear weekly pattern, with most pig movements (87% n = 20,125) occurring from Monday to Thursday (Figure 2B and C). The timing of movements was associated with its purpose (P < 0.001, Figure 2B) and the producer type of origin (P < 0.001, Figure 2C). In particular, while movement to producers occurred throughout the week, movements to slaughterhouse were cyclical, with most of these movements occurring on Monday and only a few occurred from Friday to Sunday (Figure 2B). In contrast, little seasonal variation was observed for batches sent to either another producer (Figure 2D) or to slaughter (Figure 2E) from both professional (P = 0.749 and P = 0.646) and non-assured (P = 0.226 and P = 0.186) commercial producers across the 12 first month of the study period (i.e. for the entire year 2012). However, there was an obvious seasonality in movement from small producers, regardless of the movements were sent to another producer (P = 0.028) or to slaughter (P = 0.001). Indeed, there were significantly more movements to slaughter occurring in autumn (i.e., September to November) than in the rest of the year (Figure 2E, P < 0.001), while more movements to producers (Figure 2E) were recorded in both spring (i.e., March to May, P = 0.013) and summer (i.e., June to July, P = 0.012).

### Distances and location of movements

**Figure 3:**
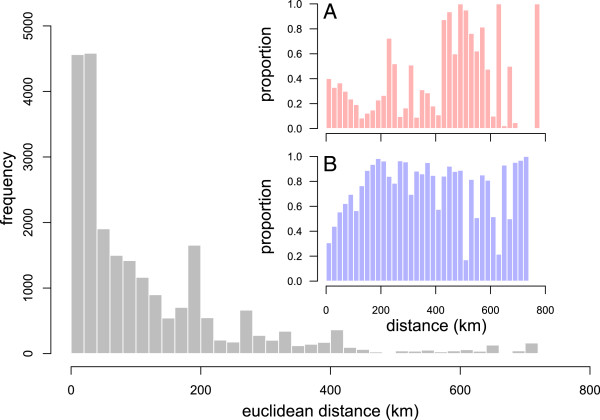
**Distribution of batch movement as a function of the Euclidean distance between departure and destination premises.** Insert **A** shows the proportion of these movements made between farms. Insert **B** shows the proportion of these movements made with an haulage company.

**Figure 4:**
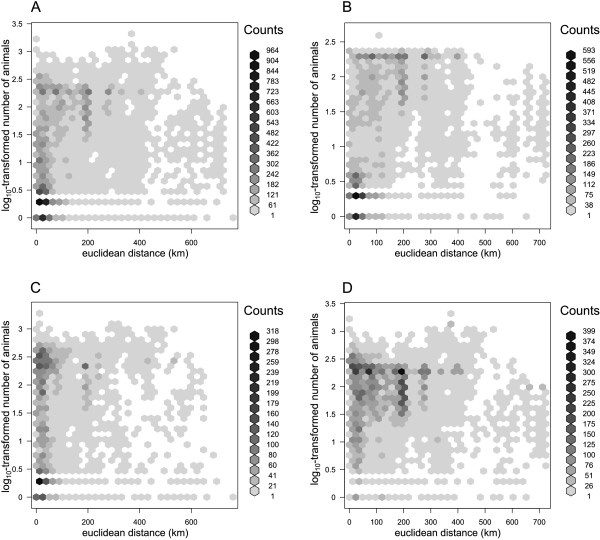
**Distribution of batch sizes as a function of the Euclidean distance travelled for each movement recorded in ScotEID between January 2012 and May 2013.** Overall distribution, including all types of departure and destination, is shown in **(A)**. Panels **B**-**D** show the distribution of batch sizes where: **(B)** only movements to slaughter, **(C)** only movements between producers, and **(D)** only movements using a haulage company have been considered. Coloured hexagonal binning has been used to illustrate the number of movements across the figure [20].

The distribution of distances travelled per batch of pigs is shown in Figure 3. The overall median distance was 65 km (Q1-Q3: 26 km – 179 km) but this differed depending on the purpose of the movement. Median distance to slaughterhouses was 95 km (Q1-Q3 35 km - 194 km) from the premises of origin, whereas non-slaughter movements were shorter (median 35 km, Q1-Q3: 15 – 96; P < 0.001; Table 1). However, movements of more than 400 km were 20% more likely (OR = 1.21, 95% CI 1.07 - 1.37, P = 0.002) to be carried out between producers than between any other types of premises (insert A in Figure 3). Figure 4 explores the relationship between the number of animals per batch and the distance travelled. Overall, there is a weak but statistically significant positive correlation between batch size and distance moved (Spearman’s rank correlation coefficient *ρ* =0.345, P < 0.001). This correlation is weakened when it is restricted to movements between farms (*ρ* =0.197, P < 0.001), but strengthened (*ρ* =0.466, P < 0.001) for movements to slaughter only.

**Figure 5:**
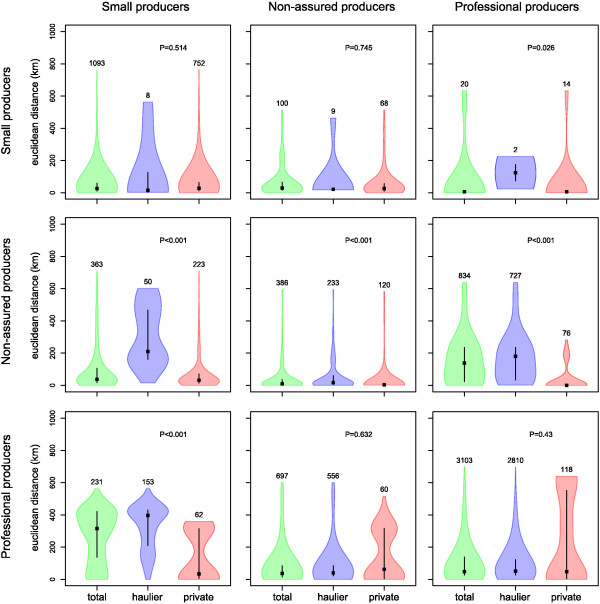
**Distribution of Euclidean distances between producers, stratified by their producer types and their usage of a registered haulage company.** Rows and columns represent departure and destination, respectively. For each panels, the three shapes represent the distribution of distances computed over all movements (“total”), all movements using a vehicle from a haulage company (“haulier”), and all movements using the vehicle from either the departure or destination (“private”). The thickness of the shapes indicates the probability density of the data, whereas the black box and solid line within each distribution indicate the median and interquartile range of the observed distribution, respectively. Numbers on the top of each distribution indicates the number of recorded movements. The P-value of the Mann–Whitney test comparing the distances recorded for movements using a haulier with those using producers’ own vehicles is also shown.

Overall, small producers tended to move pigs in significantly smaller distances than other commercial producers, whether it was for slaughtering their animals (median = 33 km, Q1-Q3: 18 km-59 km; P < 0.001) or for other purposes (median = 27 km, Q1-Q3: 11 km-58 km; P < 0.001; Table 1). However, pigs from small producers may still travel long distances; with the maximum distance (593 km) similar to that of commercial farms (~720 km) (Table 1). The distribution of distances travelled by each batch between producers as a function of the producer type of destination and departure is described in Figure 5. Most distances between producer types are small; median distances ranged from 6.4 km (from small producers to professionals, Q1-Q3 6.4 km-11 km) to 48 km (from professionals to professionals, Q1-Q3 27 km-139 km). In contrast, pigs moving from professionals to small producers (median = 315 km, Q1-Q3: 137 km – 421 km) and pigs moving from non-assured to professional producers (median = 138 km, Q1-Q3: 23 km – 235 km) appeared to travel longer distances in comparison to the others.

**Figure 6:**
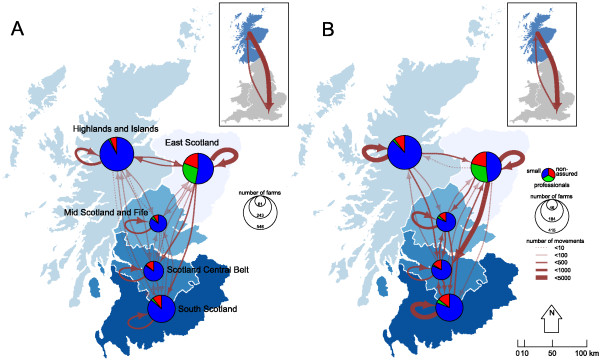
**Flow of pig movements as recorded in ScotEID between January 2012 and May 2013.** Connectivity within and between Scottish regions through pig movements where: **(A)** all movements to slaughter have been discarded from the analyses, and **(B)** only movements to slaughter have been considered. Inserts in **A** and **B** show the flow of cross-border movements between Scotland and the rest of Great Britain for non-slaughter movements and movements to slaughter, respectively. Each region in A and B is represented by a pie chart showing the number of producers involved in pig movements as a function of their producer types. The size of the pie chart indicates the total number of producers involved in pig movements either as departure or destination **(A)**, or as departure only **(B)**. Arrows represent the movement, as well as its direction, of pigs. The width of the line indicates the number of batches moved between premises types. Shaded areas in A and B indicate the boundaries of each region considered in this study.

The interconnectivity of Scottish regions via pig movements is described in Figure 6. When excluding all batches of pigs sent to slaughter, movements within regions dominated the flow of pigs in Scotland (Figure 6A); with “East Scotland”, the region with the largest number of commercial producers, showing more intra-regional movements than expected given its number of farms. Excluding “East Scotland”, there was a strong linear relationship between the number of movements carried out within each region and their number of farms (*β* = 1.2533, SE = 0.2135, P = 0.028, *R*^2^ = 0.92). Table 3 shows the E-I index for premises regrouped per region, once all movements to slaughter have been excluded. In concordance with Figure 6A, producers in “Highlands and Islands” and in “East Scotland” prefer to move animals within the same region, whereas those in the rest of Scotland were not discriminatory.

Although more batches are moved across Scotland for slaughter than for non-slaughter purposes (Figure 6B), the importance of within-region flow still remains. Indeed, when looking at the assortativity of movements to slaughter only (Table 3), slaughterhouses in “Highlands and Islands”, “South Scotland” and “East Scotland” significantly tend to receive pigs from local pig producers, while slaughterhouses in “Scotland Central Belt” tend to receive pigs from other regions. In contrast, producers in “Highlands and Islands” prefer to send their pigs to slaughterhouses within the same region (Table 3), whereas those in the rest of Scotland were not discriminatory.

There was nearly a 7-fold difference between movements going South and North across the border between Scotland and England, regardless of the purpose of the movement (Table 1). Of the 3144 cross-border movements to slaughter, 89% (n = 2802) went South (Figure 6B); similarly, 84% (1049/1246) of cross-border pig movements to farms, markets or show grounds went also South (Figure 6A). These cross-border movements represented 6%, 24% and 22% of all movements to slaughter from small, non-assured and professionals producers, respectively. Similarly, 11%, 21% and 11% of the non-slaughter movements from small, non-assured and professionals producers crossed borders, respectively. Among the 987 cross-border movements to farm, markets and show grounds departing from Scotland, 524 (53%) and 353 (36%) originated from “East Scotland” and “South Scotland”, respectively; with both sending pigs to “Yorkshire and The Humber” (22% and 38%), “North East England” (33% and 31%) and “North West England” (16% and 17%). In addition, 26% of cross border movements from “East Scotland” had as destination the “East of England”. Concomitantly, 119 (60%) of the 199 cross-border movements originating from England and Wales originated from these northern English regions (i.e. “North East England”, “North West England” and “Yorkshire and the Humber” together), with 53 (27%), 99 (50%) and 24 (12%) of the 199 cross-border movements arriving in “East Scotland”, “South Scotland” and “Scotland Central Belt”, respectively.

### Movements by hauliers

There were 13,952 (60%) movements that reported the use of a haulage company (Table 2), whereas 6723 (29%) reported the use of personal vehicles (either from the seller or the buyer of the pigs). The remaining 2494 (11%) movements did not show any records of using a haulier or not. Overall, 50 unique registered haulage companies and three stock improvement companies were used, with a median of 28 movements (Q1-Q3: 4–179) per company over the whole period. Of all movements using haulage, 13,631 (97.7%) departed from a producer, the remaining 321 departed from markets (259, 1.9%), and ferry collection centres (62, 0.4%). In contrast, a producer was the destination for only a third (4610) of these movements. The remaining 9342 movements had slaughterhouses (8448, 60.6%), markets (892, 6.4%) and ferry (2, <0.1%) as a destination (Table 2).

Overall, 501 farms reported the use of at least one haulage company, among which 187 (37%) both received and sent pigs; and 152 (30%) and 162 (32%) were only sending and receiving pigs, respectively. Overall, farms used a range of 1 to 12 unique haulage companies to send pigs (mean 2.8, median 1.0, Q1-Q3 = 1.0 – 5.0). When movements to all gathering places were excluded, farms used a range of 1 to 7 hauliers (mean 1.5, median 1.0) to send pigs to other farms, with professional producers using significantly more unique haulage companies (mean = 1.7, median = 1.0) than small producers (mean = 1.2, median = 1.0, P = 0.003). In contrast, professional and non-assured (mean = 1.4, median = 1.0, P = 0.076) producers used a similar number of unique haulage companies to send pigs.

Among the 53 haulage companies recorded in ScotEID during the study period, 38 (72%) moved pigs between farms. As such, between 1 and 132 farms (mean 14.11, median 2.5) were connected by a single haulage company. It is worth noting that, among the 132 farms connected by a single haulage company, individual farms were only trading directly with 1 to 78 others (mean 12.3, median 7.0, Q1-Q3 4.0 – 20). In addition, 19 of the 38 hauliers operated within a single region, with a mean of 2.7 regions (median 1.5) and a maximum of 10 regions. Table 2 shows the proportion of movements using a haulage company between different Scottish regions. There is a clear disparity between regions: “East Scotland” had a high level, “Scotland Central Belt” and “South Scotland” had a medium level, and “Highlands and Islands” and “Mid Scotland and Fife” had a low level of haulage use.

Use of a haulier is strongly dependent on the producer type of departure. Regardless of the premises type of destination, professional producers were four times more likely to use a haulier than a non-assured producer (OR = 4.0, 95% CI 3.7 – 4.3), whereas small producers were 44 times less likely (OR 0.023, 95% CI 0.018 – 0.028) than a non-assured producer. The magnitude of these associations was amplified when movement to all gathering places were excluded. Professionals were nearly 6 times (OR = 6.1, 95% CI 5.1-7.2) more likely than non-assured producers to use haulage, whereas small producers were 104 times (OR = 0.009, 95% CI 0.006 – 0.015) less likely to use haulage than non-assured producers.

Compared to shorter distances, movements of more than 200 km were more likely to be made using hauliers (Insert B, Figure 3). The odds of using haulage increased with each log_10_-unit increase in the number of kilometres travelled (OR = 7.0, 95% CI 6.6–7.5, P < 0.001). Figure 5 shows the number and distance distribution of movement between producer types using either a haulier or own vehicles. In addition to the fact that all producer types showed some movements using private vehicles, it is clear that they behaved differently when moving animals. While movement of pigs from professionals to small producers (OR = 9.6, 95% CI 6.8 - 14) or to non-assured producers (OR = 2.6, 95% CI 1.8 – 3.5) were more likely made using private vehicle than using a haulier, the distance travelled to small producers using a haulier was significantly greater than that using other means (difference in means = 145 km, P < 0.001). Pigs from non-assured producers were more likely to be transported using hauliers when moving to professional producers (OR = 4.9, 95% CI 3.6 – 6.8) in comparison to another non-assured producer, whereas movements to small producers were more likely made using private vehicles (OR = 8.6, 95% CI 5.9 – 13). However, distances travelled using hauliers were significantly greater than using private vehicles for all departure types (difference in medians = 174 km, 4.9 km and 75 km for movements to small, non-assured and professional producers, respectively; P < 0.001). Finally, small producers used more often hauliers to move pigs to non-assured (OR = 12, 95% CI 4.5 – 34) and professional (OR = 14, 95% CI 1.8 - 64) producers than when moving pigs to other small producers.

## Discussion

This is the first study to use data from a national electronic system for pig traceability to describe, in detail, pig movements occurring between different types of producers including backyard and hobby producers. This study had a clear objective to provide better information on the level of interaction between the different actors, commercial and non-commercial, in the Scottish swine industry. The recent epidemic of African swine fever in the eastern European region has highlighted the involvement of non-commercial swine producers in the spread of swine fevers via animal movements [21] and the threat that they may pose to both national and regional swine industries [22]. Therefore, a better understanding of these relationships is crucial for identifying likely routes of disease incursion and transmission prior to disease detection, and identifying weaknesses in biosecurity, surveillance and contingency planning. Such knowledge is a key component in the development of a representative, and therefore more robust, biosecurity and surveillance planning which would secure the resilience of the Scottish swine industry to any incursion of exotic notifiable diseases.

Movement of live pigs leading to disease transmission by direct contact is an important route of transmission for swine pathogens [18,23]. Therefore quantifying the movement patterns of pig keepers, including small producers, provides valuable information to inform disease models and risk assessments. However, other transmission routes can also be important, depending on the pathogen. Transmission via fomites is another important route of disease transmission for a number of pathogens [18,24], and could include transmission via vehicles or personnel that visit more than one type of pig unit, potentially providing additional links between commercial and non-commercial units. Spread of infectious disease, such as classical swine fever or porcine reproductive and respiratory syndrome virus, via the movement of contaminated semen have been demonstrated in Europe [25,26]. However, there is little information available on interactions between producers through semen, despite the potential importance of semen in the spread of disease within and between countries. Whilst commercial pig semen producers maintain high biosecurity to reduce the risk of pathogen transfer, it is not known how common use of semen is between smaller producers, who may use other sources of semen. Swill feeding is also thought to have been responsible for the initial incursion, and in some cases ongoing spread, in outbreaks of swine diseases, including classical and African swine fevers and foot-and-mouth disease [22]. However, swill feeding is not currently permitted in UK. Whilst wild boar play a role in transmission of swine pathogens in other areas, the wild boar population in Scotland is thought to be too low to maintain disease, although wild boar could be responsible for local transmission. Knowledge of all important transmission routes is critical for preparing for future incursions of exotic notifiable diseases and for the creation of accurate and reliable computerised disease outbreak models that may be used to inform policy. Quantification of pig movements conducted in this study provides detailed and valuable information on one transmission route, but also highlights a number of other transmission routes for which there are important knowledge gaps, particularly around the potential role of small scale pig producers.

Historically, characterisation of the interactions between commercial and non-commercial swine producers has been difficult and is rare in the scientific literature. Many previous studies were reliant on questionnaire surveys which invariably suffer from reporting bias and/or non-representative sampling of the study population [4,5,27]. Here, we used a national movement database where information on movements is electronically reported by farmers themselves. Under Scottish [16] and European legislation [17], all pig keepers moving animals are required to provide information on pig movements within Scotland, and between Scotland and England, as well as details on transportation type and location of all deliveries. As such, ScotEID provided detailed information on pig movements in Scotland at sufficient resolution to allow analyses. A potential limitation of using self-reported data is that some movements may not be reported (reporting bias) [28-31] or erroneously labelled (misinformation bias) [31,32]. Misinformation bias is likely to be minimal in ScotEID because of cross-referencing procedures which are in place to assure accurate reporting; farmers are indeed required to (1) be registered to the national pig keeper database and enter existing postcodes and premises identifier prior to reporting movements, and (2) report any movement that they receive. Although reporting bias may also be (at least partly) minimised by these cross-referencing procedures, underreporting still remains possible and therefore result in an underestimate of the number of movements that occur between premises, particularly between those that do not keep pigs for commercial purposes. It was also difficult to define the production type for all farms trading pigs in ScotEID. Although pig producers that belong to a health quality scheme could be differentiated as a function of their production system (i.e. genetic supplier, farrow-to-finishers, feeders, weaners or breeders), this was not possible with certainty for the remaining producers. As a consequence, the structural level of businesses (i.e. herds associated in so-called 'pyramid' breeding organisations) that policy targets when facing emergency could not be defined nor extracted from other readily available databases for Scotland. Instead, after discussion with the Scottish swine industry, producers were regrouped into three categories according to their pig population size, movement activity and health quality assurance scheme membership. These categories were considered the most representative of different production types, and were also likely to reflect characteristics such as the level of biosecurity carried out on farm. Indeed, the degree of biosecurity on livestock farm has been shown to be related to (1) management practices and size [5,6,33-35], and (2) quality health assurance scheme membership [36]. This information is particularly relevant to disease transmission and response planning exercises [6]. However, providing more details on premises production types and the purposes of movements (for example by including additional question in the movement databases register) would drastically increase the potential of such analyses to inform policy.

In this study, although the majority of movements were to a slaughterhouse, we found that a high connectivity between commercial and non-commercial swine producers still remains when considering non-slaughter movements, particularly between small and non-assured producers (Figure 1). This finding is in clear contrast to what has been previously considered and reported in Europe [9]; although based on a combination of (1) expert opinion and (2) published literature from countries where non-commercial pig farming is limited. Alternatively, a questionnaire survey targeting all known pig holdings in New Zealand [4] showed an outcome similar to what was found in Scotland. Nevertheless, not all movements have the same impact on the risk of pre-detection disease spread. Professional producers are responsible for the majority of the animals moved (71.9%), frequently transporting a large number of animals either for slaughter or to other premises and travelling larger distances to get their pigs marketed (Table 1, Figure 5). In contrast, Scottish small producers are characterised by a low number of movements, with a low number of pigs per batch and shorter distances travelled (Table 1, Figure 5). Thus if not detected, an incursion occurring among small producers would be limited and less likely to involve wide geographic spread but a disease incursion spreading via professional producers could feasibly generate geographically widespread epidemics through exposure of a large number of farms and animals. Given that three times more non-commercial producers move pigs than commercial ones (Table 1, Figures 1 and 6), this is consistent with results from modelling exercises showing that epidemic take-offs are unlikely to occur in the Scottish swine industry, should a highly infectious disease be introduced [32]. However, in that study, the model parsimoniously assumed that all primary outbreak farms would be reported, on average, in four weeks, regardless of the heterogeneity in the farm population size and biosecurity measures occurring on farm. While these figures on detection period are consistent to what was observed during epidemics of classical swine fever in the UK and the Netherlands [37,38], the latter only involved commercial farms where veterinary inspection are routinely carried out. However, there are likely to be characteristics of small producers, such as less regular visits by veterinarians and lower standards of biosecurity [5], which could negatively impact on the efficiency of surveillance systems, thereby allowing incursions in small producers to remain undetected for significant periods of time. In this case, the risk of pre-detection spread of disease could increase and magnify the potential of the Scottish swine industry to initiate epidemics of swine diseases.

It was interesting to note that only small producers showed seasonal variations in their movements, with more outward movement to other producers in spring/summer followed by a peak of movement to slaughter in autumn (Figure 2D and E). This pattern is likely to reflect smallholders who buy weaners in spring to finish over the summer months. Seasonal variations in small scale production have been previously observed in the Caucasus where a peak of movement to slaughter has been reported for the end of the year celebrations [39]. Quantification of this seasonal pattern is useful as movement peaks are likely to influence disease transmission and therefore be of importance when managing disease incursions or considering disease control options, particularly the impact of movement restrictions. Whilst movements to slaughter present less of a risk of onward transmission, increased movement of young pigs between producers would increase the risk of spread of any pathogens present.

According to our results, both small and professional producers demonstrated tendencies to trade animals with farms of similar features (Table 3). This finding suggests that the likelihood of spillover of disease from small producers into the commercial pig sector is low. This finding was surprising. Although we anticipated that professional producers (who adhere to quality assurance scheme guidelines on risks associated with animal trading) would trade with other professionals, we expected small producers to buy pigs indiscriminately from commercial and non-commercial producers, because they do not produce sufficient pigs to enable the sustainability of their production system. We therefore anticipated that small producers would show a high degree of interaction with other producer types. Although such a hypothesis may partly explain the observed difference between the selling (E-I Index of −0.79) and buying (E-I Index of −0.26) behaviour in this sector (Table 3), this clearly did not outweigh the importance of within-type trade. In contrast, non-assured commercial producers showed a high degree of trade (selling and buying) with other producer types (Table 3). Together with similar level of production outputs but possibly reduced biosecurity standards than professional producers, it is therefore plausible that non-assured producers may represent a bridge between the commercial and non-commercial sector of the industry, potentially allowing undiagnosed pathogen to spread throughout the industry. To test this hypothesis, network analysis can be applied to further explore the connectivity between producers via the movement of pigs [10,11,13,14], identify characteristics that may increase the influence of some producers on the spread of pathogen [10,11] and whether this influence is permanent or restricted in time [40]. Such an analysis will be the subject of another paper.

The role of livestock haulage in the spread of pathogens within and between sectors of the industry is likely to be important since animal transporters may act as fomites and spread pathogens onto farm premises and their surroundings [19,41-43]. In the Scottish swine industry, haulage is mostly used by commercial professionals and, to a lesser extent, by non-assured commercial producers, whereas small producers rarely use hauliers for transporting their pigs (Figure 5). We also showed that haulage has the potential to increase the interconnectivity between farms within the commercial sector of the industry, without showing any evidence of direct exchanges of animals. Indeed, during the study period, a single haulage company may have connected 132 farms, of which, only a fraction were directly trading animals. These results are relevant when preparing for future incursions of exotic notifiable diseases as this degree of connectivity could increase the rate of pathogen dissemination across both the Scottish and GB swine industries. Although regulations are in place for assuring that livestock haulage vehicles are regularly cleansed and disinfected in an attempt to limit the risk of widespread dissemination of pathogens [44,45], effective cleansing and disinfection can be difficult to achieve, both logistically [46-48] and in terms of compliance [49]. Together with improving disease awareness and biosecurity knowledge among producers and haulage companies, ensuring that procedures are optimised to reduce the risk of transmission via contaminated haulage vehicles is of high importance, particularly within the commercial sector.

During the study period, half of the haulage companies that moved pigs between producers operated within a single region. This result is in agreement with the local clustering of haulage use observed in a survey of quality assured producers in GB [7] and may create some geographical limitations to disease spread. In contrast, nearly 20% of movements to slaughter were sent across the border to England, especially in regions close to the border and in the East of England (Figure 6B, Tables 1 and 2). Although this pattern of pig movements to England highlights the reduced capacity of the Scottish slaughterhouses to process pigs, particular attention should be paid to its implication in the spread of diseases between England and Scotland. Increased cross-border movements of live animals and vehicles between England and Scotland is likely to significantly increase the risk of disease incursion from one country to another, either directly or due to returning vehicles [19,50]. From the point of view of livestock disease regulation agency, surveillance should therefore be modulated as a function of the frequency of incoming movements and the density of movements within Scotland. As such, targeting producers in “South Scotland” and “Scotland Central Belt”, as the likely entry point from cross border movements would likely be the most cost-efficient surveillance strategy for cross-border incursions. In contrast, farms in “Mid Scotland and Fife” may be tagged with a lower priority as it seems relatively isolated from farms in the other regions in terms of trade (Figure 6).

## Conclusions

This study has shown that the non-commercial and the commercial sectors of the Scottish swine industry coexist, showing a level of connectivity greater than expected and characterised by relatively frequent on- and off-movements of animals. We further identified differences between producer types in several animal movement characteristics which are known to increase the risk of disease spread. Specifically, distance travelled and the use of haulage were found to be significantly different between producers and dependent on the type of the producer of origin of the pig movement. As well as providing interesting insight into the structure of the swine industry in Scotland, our findings provide crucial information which policy makers may use to develop more robust biosecurity and surveillance plans. These latter would secure the resilience of the Scottish swine industry to incursions of exotic notifiable diseases. Further research is being conducted to determine biosecurity and animal trading behaviours of small pig producers throughout Scotland. Together with our findings, results of these studies may shed some lights on the impact of the non-commercial swine sector on the spread of notifiable diseases and, ultimately, on the quality of the contingency procedures currently in place to face incursions in Scotland and GB.

## Methods

### Data management

All movement data were extracted from Scottish Livestock Electronic Identification and Traceability database system (ScotEID) which was implemented in November 2011. To avoid selection bias due to inevitable missing or non-reported movements in the early stages of implementation of the database, we restricted our analysis to all movements recorded from January 1st 2012 to May 31st 2013. We used January 1st 2012 for the start of the study period, on the basis that (1) it corresponds to the time when the previous movement database (the Scottish Animal Movement System, SAMS) recording Scottish animal movements ended (i.e. November 2011), and (2) there has been a stabilisation of the movement pattern since December 2011.

Due to the nature of the swine industry, the database provides a comprehensible picture of all movements of pigs in Scotland, and between Scotland and the rest of GB, at a batch rather than individual pig level. As such, each movement record reports the County Parish Holding (CPH) identifier and postcode for departures and destinations, the number of animals involved, the date of the movements as well as information about any haulage company that may be used for transport. Details of premises type for departures and destinations are recorded in the movement database, allowing slaughterhouses, markets, show-grounds and ferry collection centres to be differentiated from agricultural holdings. Note that all markets recorded in ScotEID operate as auctioneers holding dedicated sales/collections of pigs for onward consignment to a slaughterhouse, also named “red markets”. Collections of animals that are destined to be slaughtered are therefore regularly carried out in these markets, but remain separated from the other activities of such premises, particularly activities dedicated to sales of pigs between producers. Indeed, red markets in Scotland may sometimes operate as normal livestock auction markets.

Through the CPH identifier, the ScotEID movement database was linked to several other databases in order to obtain information on the total number of pigs and sows (2011 Scottish Agricultural Census, the 2010 GB agricultural census, and the 2013 Quality Meat Scotland (QMS) register) and geographic coordinates (2010 Animal Movement Licensing System (AMLS)). All premises with an unknown CPH identifier from these databases were cross-checked against the 2013 pig keeper register, which records all holdings keeping pigs in GB and is owned by the Animal Health and Veterinary Laboratories Agency (AHVLA). Of the 494 premises that were not recorded in any of the agricultural census databases used in this study, 244 were also not recorded in the pig keeper register. In order to be as inclusive as possible, and not exclude (most likely non-commercial) producers from our study, premises that were reported with an unknown CPH identifier (either as a destination or a departure of a movement) were retained in the study if there was a corresponding valid postcode. In total, 20 premises were removed from the analysis, which corresponded to a limited number of movements (n = 224).

### Pig producers

Pig producers were classified according to their pig population size, movement activity and the health quality assurance scheme membership:

1. “Small pig producers”: agricultural holdings with an unknown number of pigs; or less than five sows, and/or less than 10 finishers; and showing no records of movements with more than 50 pigs within the study period.

2. “Non-assured commercial producers”: agricultural holdings with more than five sows and/or more than 10 finishers; or showing records of movements with more than 50 pigs during the study period, but do not belong to a quality health assurance scheme from QMS or Red Tractor, the main British assurance schemes.

3. “Professionals”: agricultural holdings with more than five sows and/or more than 10 finishers; or showing records of movements with more than 50 pigs during the study period but also belong to a quality health assurance scheme from QMS and/or Red Tractor.

### Haulier data

Details of haulage companies are recorded in the movement database, allowing identification of whether movements were carried out directly by the seller or the buyer of the pigs or using a third party. In this study, the term “haulage company” refers to a registered haulage company or a registered stock improvement company (i.e. genetic supplier moving live pigs for reproduction purposes). Details for each haulage company were collected through the QMS haulage register and http://Yell.com.

### Regions of Scotland

The flow of animals between geographical regions in the UK has been previously described by grouping considered premises into their NUTS1 (Nomenclature of Units for Territorial Statistics level 1) region as a proxy for the spatial structure of pig farm density and animal species population in the UK [7]. Although this approach is appropriate for premises in England and Wales, Scotland remains undivided. In this study, we allocated the counties of the Scottish mainland into five regions, roughly corresponding to where animals are and the production structure of the Scottish swine industry: “Highlands and Islands”, “Mid Scotland and Fife”, “Scotland Central Belt”, “South Scotland” and “East Scotland” (Figure 6A).

### Descriptive analyses

Where appropriate, Mann-Witney *U*-test and Chi-squared test were used to test for homogeneity in distribution and frequency between populations, respectively.

In this study, all geographical distances between premises correspond to Euclidian distances, expressed in kilometres, and calculated using Pythagoras’ theorem.

### Calculation of the E-I index

To evaluate the assortative nature of pig movements between types of pig producers, and between regions, we calculated the External–internal (E-I) index as in Smith et al. [7], such as:

$$E‒I\;\mathrm{index}=\frac{\mathrm{EL}-\mathrm{IL}}{\mathrm{EL}+\mathrm{IL}},$$

where *EL* and *IL* are the number of between and within group movements, respectively. As such, the E-I index measures the tendency for movements to occur either within or between groups: a value close to −1 would indicate that the majority of movements occur within a group, whereas values close to +1 indicate that most movements occur between groups [51]. Originally, the E-I index is only concerned with any connection between members and, thus, ignores the direction of the connection between group members [52]. However, movement pattern may change given the movement was received or sent. In addition, it may also change from one week to another. To explore how such a measure may vary over time and movement direction, we differentiated movements that occurred in and out of groups, as well as for every full weeks (n = 73) of the study period. In total, 146 sets of E-I indices were computed. Resulting distributions informed then on the range of possible E-I index observed for each given group. For the purpose of this study, we considered that the tendency for movements to occur either within or between groups is significant if the confidence envelop around the mean weekly E-I index, as defined by ±2SD, does not overlap zero.

Data were manipulated using MSAccess and R statistical software version 2.15.2 (R Development Core Team: 2012) and analysed using R statistical software.

## Competing interests

We declare that none of the authors at the time of the study or preparation of the paper have any competing interests that could influence or bias the content of this paper.

## Authors’ contributions

TP designed and conducted the analysis and wrote the manuscript. LAB participated in the design of the study and contributed to final manuscript. CCG provided information on the Scottish swine industry and contributed to final manuscript. HKA, GJG and MEJW contributed to final manuscript. All authors read and approved the final manuscript.
